# Beyond inflammation: exploring CAR-based cell therapy for fibrostenosing Crohn’s disease

**DOI:** 10.3389/fimmu.2026.1967353

**Published:** 2026-10-30

**Authors:** Yicheng Sun, Qiwen Chen, Chuyue Zhong, Yuxin Chen, Rui Zhao, Xixi He, Xinli Mao, Jiacheng Li, Zhenqiu Xing, Fang Wu

**Affiliations:** 1 Department of Gastroenterology, The First Affiliated Hospital of Wenzhou Medical University, Wenzhou, Zhejiang, China; Central Laboratory, The First Affiliated Hospital of Wenzhou Medical University, Wenzhou, Zhejiang, China; 2 The First Clinical College, Wenzhou Medical University, Wenzhou, Zhejiang, China; 3 Fourth Department of Liver Disease, Beijing You’an Hospital, Capital Medical University, Beijing, China; 4 Department of Gastroenterology, Taizhou Hospital of Zhejiang Province Affiliated to Wenzhou Medical University, Linhai, China; 5 Department of Neurosurgery, The First Affiliated Hospital of Wenzhou Medical University, Wenzhou, China; 6 Key Laboratory of Laboratory Medicine, Ministry of Education, School of Laboratory Medicine and Life Science, Wenzhou Medical University, Wenzhou, China

**Keywords:** chimeric antigen receptor, Crohn’s disease, fibrostenosis, immune cell therapy, intestinal fibrosis

## Abstract

Fibrostenosing Crohn’s disease (FSCD) is driven by chronic inflammation, fibroblast activation, extracellular matrix (ECM) deposition and tissue remodeling. Current management includes medical treatment to control inflammation, endoscopic interventions, and surgery to relieve clinically significant strictures. Chimeric antigen receptor (CAR)-based cell therapy represents an emerging research direction that may enable targeted intervention against selected cell populations implicated in fibrogenesis. Unlike approaches focused primarily on inflammatory control, CAR-based strategies can be engineered to recognize selected cellular populations, offering a potential means of directly targeting cells involved in fibrotic remodeling or modulating the local profibrotic microenvironment. By integrating insights from intestinal pathobiology with evidence from other fibrotic settings, we assess candidate cellular targets, therapeutic modalities, and CAR-cell platforms, and consider how these choices should be aligned with lesion accessibility, disease stage, therapeutic objectives, and safety requirements. We further discuss the translational challenges of CAR-based approaches for FSCD.

## 1 Introduction

Fibrostenosing Crohn’s disease (FSCD) is a complication of Crohn’s disease (CD) involving chronic transmural inflammation, excessive extracellular matrix (ECM) deposition, and structural remodeling of the intestinal wall, which contribute to bowel-wall thickening and luminal narrowing. FSCD can progress to intestinal obstruction and frequently coexists with penetrating disease. In addition, malignant transformation may occur at the site of stricture. The diagnosis of FSCD must rely on cross-sectional imaging including computed tomography (CT), magnetic resonance imaging (MRI), and intestinal ultrasound (IUS), or endoscopic evaluation. Diagnostic criteria include bowel wall thickening, luminal narrowing, and pre-stenotic dilatation on cross-sectional imaging, or inability to pass an adult colonoscope through the strictured segment (1).

Currently, clinical management of FSCD relies on anti-inflammatory medications, endoscopic therapy, and surgical intervention. Pharmacological treatments, including biologics and small-molecule drugs, primarily targets inflammatory pathways and is ineffective against fibrosis independent of inflammation (2). For fibrotic strictures without active inflammation, endoscopic therapy or surgical intervention is indicated, yet these invasive approaches are associated with substantial risks of symptom recurrence and re-intervention. Therefore, the identification of specific therapies that directly target the fibrostenotic process represents a major research priority in the field of CD.

Chimeric antigen receptor (CAR)-based engineered immune cell therapy has expanded from oncology to the treatment of fibrotic diseases, with preclinical studies in non-intestinal organs demonstrating that engineered immune cells can target fibrosis-associated cell populations or modulate local immune responses (3). Translating this approach to FSCD requires consideration of disease stage, therapeutic objectives, CAR design, and the selection of appropriate cell platforms and molecular targets, and the available evidence needs to be interpreted according to its relevance to FSCD. In this review, we summary the evidence supporting CAR-based cell therapy for FSCD and examine the safety, delivery, and clinical assessment challenges that must be addressed before this approach can be translated to patients.

## 2 CAR-based cell therapy in fibrotic diseases

Fibrosis is characterized by excessive deposition of ECM, leading to disruption of organ architecture and functional failure (4). Conventional antifibrotic therapies have shown limited efficacy, and CAR-based engineered immune cell therapy represents an emerging approach that enables engineered cells to recognize selected cellular targets and exert cytotoxic or modulatory effects, potentially opening new avenues for the treatment of fibrotic diseases.

### 2.1 Key cell types and their roles in fibrosis pathogenesis

This section reviews the core mechanisms driving fibrosis, organized by key cell types (**Figure 1**). Although fibrosis affects different organs, the core cell types and mechanisms driving fibrosis are highly conserved across tissues (5). Understanding these shared mechanisms provides the theoretical foundation for developing strategies targeting these cells to treat fibrotic diseases.

**Figure 1:**
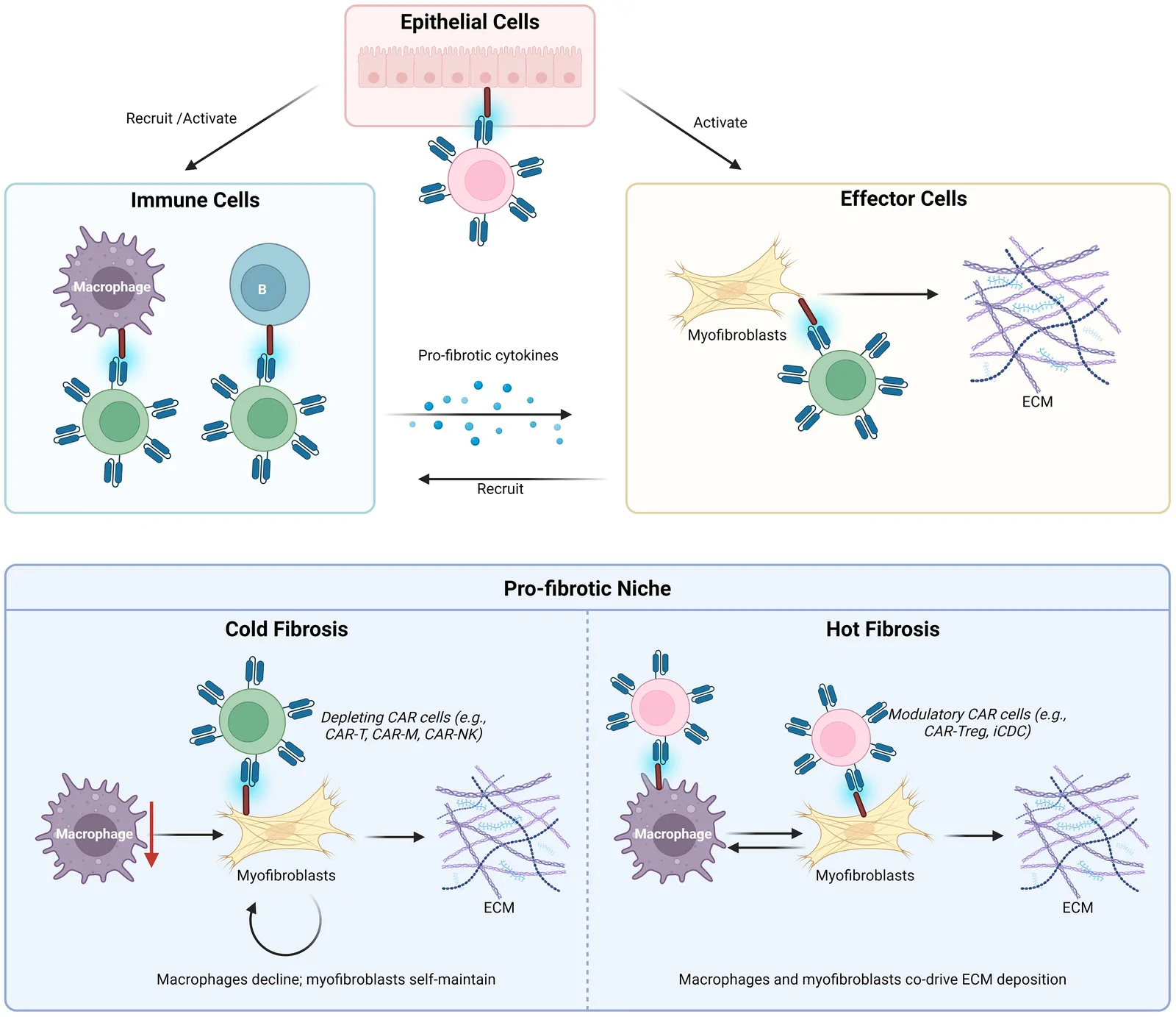
Core cellular mechanisms and the pro-fibrotic niche in organ fibrosis. Created with “BioRender.com”.

#### 2.1.1 Epithelial cells

Similar to the initiation mechanisms of normal wound healing, pathological organ fibrosis begins with epithelial cell injury (5). Upon cellular injury, the released damage-associated molecular patterns (DAMPs) are promptly sensed by pattern recognition receptors on immune cells such as macrophages, activating downstream signaling pathways including NF-κB, which in turn induces the production and release of pro-inflammatory cytokines, ultimately triggering the inflammatory response (6). Concurrently, injured epithelial cells secrete profibrotic factors such as transforming growth factor-beta (TGF-β) and interleukin (IL)-33, directly activating tissue-resident mesenchymal cells and driving their phenotypic conversion toward myofibroblasts (5). In addition, chemotactic signals emanating from injured epithelial cells direct the extravasation of circulating monocytes into the injured site (7). Epithelial-mesenchymal transition (EMT) has also been proposed as a potential pathway linking epithelial cells to fibrogenesis, though this mechanism remains controversial (8, 9).

These findings suggest that epithelial cells may contribute to fibrogenesis primarily through injury-associated inflammatory and paracrine signals rather than solely through direct conversion into matrix-producing cells.

#### 2.1.2 Stromal cells

##### 2.1.2.1 Fibroblasts and myofibroblasts

According to the review by Younesi et al. (5), fibroblast activation and their functional state transition toward myofibroblasts are central to excessive ECM deposition and tissue remodeling during fibrosis. Following tissue injury, various tissue-resident mesenchymal cells undergo activation and acquire functional states required for repair. Among these, the myofibroblast is a functional state characterized by high expression of alpha-smooth muscle actin (α-SMA), with its primary function being the generation of contractile force and the driving of tissue contracture. Meanwhile, the extensive synthesis and secretion of ECM is mainly carried out by fibroblast subsets expressing markers such as CTHRC1. Although the etiology and clinical manifestations of fibrosis vary across different organs, activated fibroblasts persistently exist in fibrotic diseases and contribute to ECM deposition and tissue contracture observed in all affected organs.

##### 2.1.2.2 Other stromal cells

Endothelial cells exhibit marked dysfunction in organ fibrosis, manifesting as morphological abnormalities, impaired barrier function, and upregulation of activation markers (10). Under pathological stimuli, endothelial cells can acquire a mesenchymal phenotype through endothelial-to-mesenchymal transition (EndMT) (11), while activated endothelial cells can directly activate fibroblasts through paracrine secretion of TGF-β1 and connective tissue growth factor (CTGF) (12, 13), and contribute to the amplification of inflammation by enhancing immune cell adhesion (10). Pericytes and vascular smooth muscle cells serve as important precursor cell sources for myofibroblasts (5, 11, 14).

Collectively, these findings identify stromal cells as important participants in fibrogenesis and highlight the heterogeneity of the stromal compartment. Endothelial cells, pericytes, vascular smooth muscle cells, and fibroblasts contribute through distinct but interconnected mechanisms.

#### 2.1.3 Immune cells

Activated stromal cells recruit and activate immune cells through the secretion of chemokines and cytokines, and immune cells in turn can further activate stromal cells by secreting mediators such as TGF-β, forming a positive feedback loop (15).

##### 2.1.3.1 Macrophages

Within the fibrotic microenvironment, macrophages represent a key immune cell population. Under the influence of macrophage colony-stimulating factor (M-CSF) in the microenvironment, monocyte-derived macrophages can maintain their profibrotic phenotype (16). These macrophages not only drive fibroblast activation and collagen deposition through growth factors such as platelet-derived growth factor (PDGF) and TGF-β, but also remodel the transcriptional profile of fibroblasts via the secretion of IL-1β, tumor necrosis factor-alpha (TNF-α), and other mediators, inducing them to acquire a pro-inflammatory phenotype (17–19). Furthermore, vitronectin secreted by activated macrophages can be integrated into the ECM network and, by binding to integrin αvβ5 on the fibroblast surface, activate Src kinase signaling, thereby enhancing their activation state and promoting ECM deposition (20).

##### 2.1.3.2 T cells

T cells are the core effector cells of adaptive immunity and play complex and critical roles in fibrosis. Based on T cell receptor type and function, they can be further divided into multiple subsets.

Among CD4+ T cells, T helper 1 (Th1) cells have traditionally been considered antifibrotic, as Th1-derived interferon-γ (IFN-γ) inhibits fibroblast collagen synthesis and promotes matrix metalloproteinase (MMP)-mediated ECM degradation (21). In autoimmune hepatitis, however, Th1 cells promote hepatocyte apoptosis and inflammation through IFN-γ and TNF-α production, indirectly facilitating fibrosis progression, suggesting that their role is tissue-specific (22). T helper 2 (Th2) cells are a well-recognized profibrotic subset whose secretion of IL-4 and IL-13 directly activates fibroblasts and induces collagen deposition. T helper 17 (Th17) cells promote fibrosis through the secretion of IL-17A. Regulatory T cells (Tregs) generally exert antifibrotic effects by secreting IL-10 to suppress effector T cells, yet under certain circumstances they may also promote fibrosis through the production of TGF-β, among other mechanisms (23). This discrepancy may stem from an imbalance between the immunosuppressive and tissue repair functions of Tregs (24).

The role of CD8+ T cells in fibrosis also exhibits organ- and subset-specificity. In organs such as the heart, lung, and skin, they primarily exert profibrotic effects, whereas in the kidney, they exert antifibrotic effects through IFN-γ (23, 25, 26).

##### 2.1.3.3 B lymphocytes (B cells)

B cells primarily play a profibrotic role in most organ fibrosis, with mechanisms including the production of pathogenic antibodies, secretion of pro-inflammatory/profibrotic cytokines, antigen presentation to activate T cells, and even direct collagen production (27, 28). Therapeutic strategies targeting B cells have demonstrated antifibrotic potential in animal models (27–29).

The profibrotic or antifibrotic effects of immune cells cannot be inferred solely from their lineage identity. As illustrated by the context-dependent effects of Th1 cells, CD8^+^ T cells, and Tregs, the same broad cell population may exert different effects depending on its functional state, tissue environment, and stage of disease. For therapeutic target selection, it is therefore necessary to find a disease-specific and stage-specific rationale for selecting the target population or function and distinguish the presence of an immune-cell population from evidence that the population causally sustains profibrotic signaling.

#### 2.1.4 The fibrotic niche

As injury persists or profibrotic signals are sustained, a profibrotic niche is established (30). This niche is composed of parenchymal cells such as fibroblasts and endothelial cells, infiltrating inflammatory cells such as macrophages, together with a specialized ECM network, soluble molecules, extracellular vesicles, and metabolites. Within this niche, increased tissue stiffness is not merely a consequence of fibrosis but a key active driver of fibrosis progression. The stiffened ECM transmits mechanical signals to fibroblasts via integrin receptors, promoting their conversion toward a myofibroblast phenotype and maintaining the activated state (31). This mechanotransduction acts in concert with biochemical signals, forming a positive feedback loop that endows the niche with self-sustaining capacity.

Within the profibrotic niche, continuous interactions exist between immune cells and stromal cells. These interactions include physical contacts and paracrine signaling. Macrophages can activate fibroblasts through two sequential contact-dependent mechanisms: acute activation via the αvβ3 integrin-Piezo1 mechanotransduction axis. In contrast, cadherin-11-mediated adhesion between macrophages and myofibroblasts supports their sustained interaction and maintains a profibrotic niche through localized TGF-β activation (32, 33). Reciprocally, activated fibroblasts can polarize into inflammation-associated fibroblasts, a cell type that further recruits immune cells through chemokine secretion while simultaneously releasing inflammatory mediators that amplify the local inflammatory response (15, 34).

The cellular composition and maintenance mechanisms of the niche can exhibit two distinct steady states depending on the mode of injury, referred to as “cold fibrosis” and “hot fibrosis” (14). Niches driven by acute injury, such as myocardial infarction (MI), are characterized by “cold fibrosis”. During the early phase of injury, macrophages infiltrate abundantly and interact with activated fibroblasts. As the disease progresses, macrophage numbers gradually return to baseline, while myofibroblasts achieve self-maintenance through autocrine loops, becoming the core driving force of fibrosis progression (14, 35). In contrast, in the “hot fibrosis” niche driven by chronic injury, myofibroblasts and macrophages co-exist persistently through mutual paracrine support, jointly driving ECM deposition (36).

These findings indicate that fibrosis may be maintained by different combinations. Consequently, identifying the dominant maintenance mechanism in a given disease context may be as important as identifying the cells initially involved in fibrogenesis. A population that contributes to early injury-associated remodeling may not remain the principal driver of established fibrosis, while persistent matrix stiffness and cell–cell interactions may continue to support pathological tissue remodeling after the initiating inflammatory response has changed. This distinction provides a biological basis for evaluating CAR-based therapies in relation to maintenance mechanisms they are designed to address and the cellular populations, rather than treating the fibrotic niche as a single, uniformly targetable entity.

### 2.2 CAR-based therapeutic approaches in fibrotic diseases

CAR-based engineered immune cell therapy is expanding from hematological malignancies to immune-related diseases, offering a potential new direction for the treatment of fibrotic diseases. Unlike approaches that solely target single signaling molecules, CAR-based cell therapy, through the target-cell depletion or modulation of target cells and the microenvironment, has demonstrated significant efficacy in various animal models of fibrosis (**Table 1**), with some strategies having entered early clinical exploration.

**Table 1:** Preclinical studies of CAR-based therapy in fibrotic diseases.

Year	Target	Disease	CAR Cell Type	Platform	Reference
2019	FAP	Cardiac Fibrosis	CAR-T	Ex vivo viral-engineered CAR-T cell	Aghajanian, H. et al. (37)
2020	uPAR	Liver Fibrosis	CAR-T	Ex vivo viral-engineered CAR-T cell	Amor, C. et al. (38)
2022	FAP	Cardiac Fibrosis	CAR-T	*In vivo* mRNA LNP	Rurik, J. G. et al. (39)
2023	CD19	Systemic sclerosis-associated interstitial lung disease	CAR-T	Ex vivo viral-engineered CAR-T cell	Avouac, J. et al. (40)
2024	uPAR	Liver Fibrosis	CAR-M	Ex vivo viral-engineered CAR-M	Dai, H. et al. (41)
2024	FAP	Cardiac Fibrosis	CAR-M	Ex vivo viral-engineered CAR-M	Wang, J. et al. (42)
2024	FAP	Cardiac Fibrosis	CAR-M	Ex vivo viral-engineered CAR-M	Gao Z, et al. (43)
2024	FAP	Liver Fibrosis	CAR-M	Ex vivo viral-engineered CAR-M	Mao, Y. et al. (44)
2025	PDGFRβ	Chronic Kidney Disease (Kidney Fibrosis)	CAR-T	*In vivo* mRNA LNP	Zhao, S. et al. (45)
2025	FAP	Cardiac Fibrosis	CAR-NK	Ex vivo viral-engineered CAR-NK	Zheng, Q. et al. (46)
2025	FAP	Cardiac Fibrosis	CAR-M	*In vivo* mRNA LNP	Du, H. et al. (47)
2025	TNC	Liver Fibrosis	CAR-M	Ex vivo viral-engineered CAR-M	Chen, K.-Z. et al. (48)
2025	FAP	Idiopathic Pulmonary Fibrosis	CAR-cTreg	Ex vivo viral-engineered CAR-T	Jiang, Y.-H. et al. (49)
2025	uPAR	Liver Fibrosis, Rheumatoid Arthritis	CAR-T	*In vivo* mRNA LNP (Cardiolipin-mimic)	Zhang, Z. et al. (50)
2025	FAP	Cardiac Fibrosis	CAR-T	Ex vivo viral-engineered CAR-T (*in vitro* study)	Li, H. et al. (51)
2025	CSPG4	Duchenne Muscular Dystrophy Cardiomyopathy	CAR-T	Ex vivo viral-engineered CAR-T	Ceraolo, M. G. et al. (52)
2025	CD248	Cardiac Fibrosis	CAR-T	Ex vivo viral-engineered CAR-T	Li, G. et al. (53)
2025	FAP	Duchenne Muscular Dystrophy(skeletal muscle fibrosis)	CAR-T	Ex vivo lentiviral-engineered CAR-T	Ferrand, M. et al. (54)
2026	FAP	Cardiac Fibrosis	CAR-Treg	Ex vivo viral-engineered CAR-Treg	Zhang, M. et al. (55)
2026	FAP	Cardiac Fibrosis	CAR-T	Ex vivo viral-engineered CAR-T	Hua, X. et al. (56)
2026	FAP	Cardiac Fibrosis	CAR-DC	Ex vivo viral-engineered immunosuppressive DC	Li, X. et al. (57)
2026	FAP	Cardiac Fibrosis	CAR-T	*In vivo* mRNA LNP	Liu, X. et al. (58)
2026	FAP	Liver Fibrosis (MASH)	CAR-T	*In vivo* mRNA LNP	Yashaswini, C. N. et al. (59)
2026	FAP	Cardiac Fibrosis	CAR-T	Ex vivo viral-engineered CAR-T;ROS-responsive Hydrogel	Cao, B. et al. (60)
2026	FAP	Liver Fibrosis	CAR-M	*In vivo* mRNA LNP (TRIM13 co-delivery)	Gao, J. et al. (61)
2026	FAP	Pulmonary Fibrosis	CAR-M	Ex vivo viral-engineered CAR-M; Viscoelastic Hydrogel	Zhang, Y. et al. (62)
2026	FAP	Liver and Lung Fibrosis	CAR-M	Ex vivo viral-engineered CAR-M; *In vivo* (myeloid-specific CAR knock-in mice); adoptive transfer of CAR-Ms or whole blood	Wang, M. et al. (63)
2026	FAP	Liver Fibrosis	CAR-T	Ex vivo viral-engineered CAR-T cell	Zhang, Y.-W.-Q. et al. (64)
2026	CD19	IgG4-related disease	CAR-T	Ex vivo retroviral-engineered CAR-T cell	Hu, J. et al. (65)

#### 2.2.1 Target-cell depletion

Target-cell depletion aims to eliminate disease-associated cell populations that actively contribute to fibrogenesis or sustain pathological tissue remodeling. This strategy has been investigated primarily through the targeting of fibroblast activation protein (FAP), platelet-derived growth factor receptor β (PDGFRβ), and urokinase-type plasminogen activator receptor (uPAR) in experimental models of fibrosis.

FAP-targeted CAR-T cells provide a proof of concept for cellular depletion of activated fibroblasts. In a mouse model of hypertensive cardiac fibrosis, FAP-directed CAR-T cells reduced the burden of activated fibroblasts and attenuated cardiac fibrosis, establishing the feasibility of targeting a disease-associated stromal population *in vivo* (37). Subsequent studies have explored *in vivo* generation of CAR-T cells, intended to improve the controllability of this strategy and overcome the limitations of conventional ex vivo manufacturing (66). FAP-targeted CAR natural killer cells (CAR-NK) and CAR macrophages (CAR-M) have also been investigated as alternative cellular platforms (42, 43, 46, 47).

Related strategies have been explored in hepatic fibrosis. In models of metabolic dysfunction-associated steatohepatitis (MASH), *in vivo* transient CAR-T cells have been investigated as a means of targeting activated hepatic stellate cells or other disease-associated populations (59). CAR-M targeting uPAR represent another approach to recognizing activated or injury-associated cells in fibrotic tissues (41). To address the limitation that a persistent pro-inflammatory microenvironment can polarize the infused CAR-M cells toward a pro-inflammatory phenotype, a combined CAR-M strategy has been developed in which FAP-directed recognition is paired with TRIM13 mRNA delivery to support an anti-inflammatory macrophage phenotype while targeting activated hepatic stellate cells (49). This design illustrates the possibility of combining target-cell clearance with functional engineering of the effector cell.

FAP-directed CAR-T cells have also been investigated in experimental pulmonary fibrosis (67). Similarly, PDGFRβ-directed strategies investigated in renal settings (45).

These studies support the preclinical feasibility of CAR-mediated depletion of disease-associated stromal populations across several organs. However, the therapeutic rationale depends not only on antigen expression but also on the functional contribution of the targeted population to disease progression and the consequences of its depletion for normal tissue homeostasis.

#### 2.2.2 Target-cell and microenvironmental modulation

Modulatory strategies seek to interfere with profibrotic processes while potentially preserving beneficial cellular functions.

One approach is to use CAR-engineered regulatory T cells to modulate local immune responses and stromal activity. FAP-targeted CAR-engineered regulatory T cells (CAR-Tregs), referred to as FCTR, have been investigated as a strategy for directing regulatory activity toward fibrotic tissue (55). This approach may limit pathological immune–stromal interactions without relying exclusively on the direct elimination of activated fibroblasts.

Engineered dendritic cells represent a complementary approach to modulating the fibrotic microenvironment. In a preclinical study of cardiac fibrosis, FAP-targeted engineered dendritic cells (iDCs) were developed to direct these cells to injured myocardium and exert antifibrotic effects through immunomodulatory molecules (57).

Although some CAR-M approaches are intended to promote the removal of fibrosis-associated target populations, engineered CAR-M cells have also been explored as a means of modulating the fibrotic microenvironment. The strategy combining anti-FAP CAR expression with TRIM13 mRNA delivery aforementioned in 2.2.1 addresses a potential limitation of CAR-M therapy. By combining target recognition with additional functional engineering, this approach illustrates how CAR-based interventions may be designed to address both the identity of the target and the local conditions that influence effector-cell behavior.

These studies illustrate several distinct ways in which CAR cells help limit ongoing fibrogenesis while preserving stromal cells that retain physiological or reparative functions by interrupting profibrotic immune–stromal signaling. CAR-Tregs may provide a means of directing regulatory activity, engineered dendritic cells may be used to alter local immune responses, and engineered CAR-M cells may combine target recognition with control of their functional state.

Importantly, target-cell depletion and target-cell or microenvironmental modulation should not be regarded as mutually exclusive strategies, nor can they be distinguished solely by CAR-cell platform. For example, CAR-M cells engineered to express anti-FAP CARs and deliver TRIM13 mRNA represent a combined approach rather than a purely depleting or modulatory intervention. Similarly, although conventional CAR-Tregs are primarily designed to mediate antigen-specific immune regulation (68), emerging evidence indicates that cytolytic regulatory T cells (cTregs) can combine immunosuppressive activity with direct target-cell killing through granzyme B- and perforin-dependent mechanisms. FAP-targeted CAR-cTregs co-expressing forkhead box protein P3 (FOXP3) have been shown to selectively eliminate myofibroblasts and suppress T-cell activation *in vitro*. In a bleomycin-induced mouse model of pulmonary fibrosis, these cells attenuated pulmonary fibrosis without detectable systemic inflammatory responses (49).

#### 2.2.3 Emerging clinical evidence

While CAR-based strategies have demonstrated antifibrotic potential in animal models, preclinical studies and clinical trials differ notably in disease focus and target selection. Compared with preclinical studies, current clinical development has largely focused on autoimmune diseases with concomitant tissue fibrosis, particularly systemic sclerosis (SSc), whereas studies specifically targeting organ-specific fibrosis are relatively scarce (**Table 2**). B-cell-directed CAR therapies, including approaches targeting CD19 or B-cell maturation antigen (BCMA), represent an important component of this clinical landscape. As of March 2026, a total of 27 eligible clinical trials of CAR-based cell therapy for fibrosis-related diseases were identified on ClinicalTrials.gov, among which studies targeting CD19 or BCMA predominate. Their rationale derives from the contribution of B cells to humoral immunity and immune dysregulation in selected autoimmune diseases. By targeting disease-associated B-cell populations, these approaches may provide a means of modifying upstream immune processes implicated in chronic inflammation and tissue injury. Direct clinical investigation of CAR-based therapies for organ fibrosis remains limited compared with the broader development of CAR therapies for malignancies and immune-mediated diseases. Regarding organ-specific fibrosis, the only study currently in the clinical pipeline is a not-yet-recruiting Phase I clinical trial (NCT07329959), which plans to employ the same FAP-targeting iDC platform as used in the aforementioned cardiac fibrosis studies for the treatment of idiopathic pulmonary fibrosis (IPF).

**Table 2:** CAR-based cell therapy clinical trials for fibrosis-related diseases registered on ClinicalTrials.gov.

Identifier	Study Title	Intervention Type	Target	Disease	Fibrosis-related Outcomes
NCT06828042	Safety and Efficacy of Universal CD19-targeting CAR-γδT Cells in Refractory Autoimmune Diseases	CAR-γδT	CD19	SSc	mRSS, CRISS
NCT07148791	Anti-BCMA-CD19 CAR-T Cell Therapy in Relapsed or Refractory IgG4-Related Disease	CAR-T	BCMA-CD19	IgG4-RD	Semi-quantitative scoring of inflammation and fibrosis in lesional tissue (Histopathology of Lesion)
NCT06733935	NKX019 in Subjects With Immune-Mediated Diseases (Ntrust-2)	CAR-NK	CD19	SSc	FVC, DLCO, mRSS, rCRISS, ILD-related outcomes
NCT07329959	CAR-DC for End-Stage IPF	CAR-DC	FAP	IPF	FVC, DLCO, pulmonary fibrosis area (quantitative CT)
NCT06792344	CD19 CAR-T in Refractory Systemic Sclerosis	CAR-T	CD19	SSc	mRSS (primary outcome), CRISS
NCT07283315	Anti-CD19/BCMA CAR-NK in Pediatric B Cell-related Autoimmune Diseases	CAR-NK	CD19-BCMA	SSc	CRISS score
NCT07473154	Controlling Hyperactive Immunity With Long-lived Lymphocytes (QEL-005)	CAR-Treg	CD19	dcSSc	mRSS, HRCT pulmonary fibrosis score, PET pulmonary fibrosis assessment
NCT06614270	Anti-CD19 IL-10/IL15 CAR-NK Cells in Refractory/Relapsed Autoimmune Diseases	CAR-NK	CD19	SSc	mRSS
NCT05085444	CD19/BCMA CAR-T for Refractory Scleroderma	CAR-T	CD19-BCMA	SSc	ORR, DCR
NCT07152223	CD19-BCMA CAR-T for Connective Tissue Diseases	CAR-T	CD19-BCMA	SSc	Responder index for SSc
NCT06544330	SYNCAR-001 + STK-009 in Severe Refractory Systemic Autoimmune Rheumatic Disease	CAR-T	CD19	SSc	mRSS, rCRISS, FVC, DLCO, HRCT ILD score
NCT06377228	TAK-007 in Refractory Lupus Nephritis or Refractory Systemic Sclerosis	CAR-NK	CD19	SSc	FVC, ppFVC, DLCO, mRSS, QILD-WL HRCT score
NCT05859997	Universal CAR-T Cells (BRL-301) in Relapse or Refractory Autoimmune Diseases	CAR-T	CD19	SSc	CRISS
NCT06347718	CAR-T Cells in Systemic B Cell Mediated Autoimmune Disease	CAR-T	CD19	SSc	mRSS, FVC, ILD progression, HRCT
NCT06685042	Anti-CD19 CAR T-Cell Therapy in Refractory Systemic Autoimmune Diseases (CATARSIS)	CAR-T	CD19	SSc	No progression of ILD (FVC)
NCT06056921	Safety and Efficacy of CD19 Targeted CAR-T for Refractory Autoimmune Disease	CAR-T	CD19	SSc	mRSS
NCT06400303	KYSA-5: Anti-CD19 CAR-T in Systemic Sclerosis	CAR-T	CD19	SSc	rCRISS
NCT06328777	RESET-SSc: CABA-201 in Systemic Sclerosis	CAR-T	CD19	SSc	CRISS
NCT07322718	Safety and Efficacy of RXIM002 in Severe Autoimmune Diseases	*In vivo* CAR-T (mRNA-LNP)	CD19	SSc	mRSS
NCT06822881	CT1190B in Moderate to Severe Refractory SLE or Refractory/Progressive SSc	CAR-T	CD19/CD20	SSc	ACR-CRISS
NCT07339540	Targeted BCMA *In Vivo* LV Injection for Recurrent or Refractory Autoimmune Diseases	*In vivo* CAR-T	BCMA	SSc	CRISS-25, FVC, mRSS, QILD, QLF imaging scores
NCT06414135	Relmacabtagene Autoleucel for Systemic Sclerosis	CAR-T	CD19	SSc	CRISS, mRSS, FVC, DLCO, HRCT
NCT06255028	CNTY-101 in Refractory B Cell-mediated Autoimmune Diseases (CALiPSO-1)	CAR-iNK	CD19	dcSSc	FVC, DLCO, ILD progression, mRSS, ACR-CRISS
NCT07193667	CAR-T in Subjects With Relapsed/Refractory Autoimmune Disease	CAR-T	CD19	SSc	EUSTAR activity index (including ILD assessment)
NCT07085104	ALLO-329 in Adults With Autoimmune Disease	CAR-T	CD19-CD70	SSc	EUSTAR activity index, ACR-CRISS
NCT06821659	Universal CAR-T Cells (UWD-CD19) Combined with Immunosuppressants	CAR-T	CD19	SSc	CRISS, mRSS
NCT07184450	BCMA/CD70-targeted CAR-T for Refractory Pediatric Rheumatic Diseases	CAR-T	BCMA-CD70	SSc	mRSS, MDAI, EUSTAR activity index, CRISS

Search performed on ClinicalTrials.gov, accessed March 2026.

Screening criteria: cell types included CAR-T, CAR-M, CAR-NK, CAR-NKT, CAR-γδT, CAR-Treg, and CAR-cTreg; diseases included specific organ fibrosis, systemic sclerosis, and other autoimmune diseases involving fibrosis; outcome measures were required to explicitly include indicators directly assessing fibrosis.

## 3 CAR-based cell therapy for FSCD

### 3.1 Current management of FSCD

In FSCD, most fibrostenotic lesions are of mixed type, with coexisting inflammatory and fibrotic components. Treatment selection requires stratified decision-making that integrates the degree of inflammation and risk factors. For strictures with active inflammation, pharmacological treatment constitutes an essential component of management, including biologics, such as TNF-α inhibitors, integrin inhibitors, and anti-IL-12/23 or anti-IL-23 agents, as well as small-molecule drugs, such as JAK inhibitors (1). However, pharmacotherapy primarily targets the inflammatory component and has limited efficacy against established, fibrosis-predominant strictures, making it difficult to reverse pre-existing fibrosis (2). For fibrotic strictures without active inflammation, or for patients in whom obstructive symptoms persist despite resolution of inflammation following pharmacotherapy, endoscopic or surgical treatment may be considered (1).

Endoscopy and surgery are the principal methods for managing fibrotic strictures but also have their limitations. Endoscopic balloon dilation (EBD) is the cornerstone of endoscopic stricture management, with primary indications including FSCD with stricture length ≤ 4–5 cm, fewer than 4 strictures, pre-stenotic dilatation < 5 cm, and the absence of deep ulcers, fistulae, abscesses, or malignancy (69, 70). Although EBD achieves a high technical success rate, its efficacy is difficult to sustain (71), and it carries risks of complications such as bleeding and perforation (70). Endoscopic stricturotomy (ES) is superior to EBD, yet its one-year re-intervention rate remains 23.5% (72). Clear indications for surgery in FSCD include the development of penetrating complications or malignant transformation, stricture length > 5 cm, or endoscopically inaccessible strictures (1). For patients with clear surgical indications, surgery can effectively relieve intestinal obstruction, but the risks of postoperative complications such as anastomotic leak, intestinal obstruction, and bleeding remain, and postoperative disease recurrence is substantial (73, 74), with 5-year recurrence rates of 44%-55% (1). Current treatment modalities for FSCD exhibit significant limitations in directly addressing the fibrostenotic process, underscoring the urgent need to explore specific therapies.

Beyond the intrinsic limitations of current treatment modalities, accurate identification of patients suitable for direct antifibrotic therapy represents another critical barrier. Currently, cross-sectional imaging techniques, including computed tomography enterography (CTE), magnetic resonance enterography (MRE), and IUS, serve as the primary tools for assessing the degree of fibrosis. In recent years, these modalities have enabled semi-quantitative or quantitative grading of fibrosis through radiomics-based models, magnetization transfer imaging, and elastography (75). A retrospective study by the STAR consortium further identified stricture length, maximal stricture wall thickness, and maximal pre-stenotic dilatation as three reproducible and measurable parameters, providing standardized tools for characterizing stricture severity (76, 77). However, these approaches are largely based on single-center, retrospective data and require prospective clinical validation. Therefore, the development of tools capable of accurately quantifying the degree of bowel wall fibrosis is a prerequisite for advancing novel antifibrotic therapies such as CAR-based approaches into clinical application.

### 3.2 Pathophysiological mechanisms of FSCD

The pathogenesis of FSCD has been systematically summarized in several authoritative reviews (78, 79). Briefly, this disease is characterized by chronic inflammation, excessive activation of intestinal fibroblasts, massive deposition of ECM and tissue remolding (80). Multiple key pathogenic cell types participate in this process (**Figure 2**). Epithelial cells, under chronic injury, highly express αvβ6 integrin, which activates TGF-β and the FAK/AKT pathway, serving as a critical upstream initiator of the fibrotic cascade. In the microenvironment of persistent chronic inflammation and progressing fibrosis, macrophages polarize toward a profibrotic phenotype, becoming the principal source of profibrotic factors such as TGF-β, and can directly contribute to ECM production through macrophage-to-myofibroblast transition (MMT), thereby exacerbating fibrosis. Th17 cells and ILC3s can contribute to fibrosis through the secretion of IL-17 (81). Resident-like memory B cells tend to directly differentiate into plasma cells without undergoing secondary germinal center selection and can activate fibroblasts through paracrine signaling and promote fibrosis in FSCD (82). Activated fibroblasts are the core effector cells of ECM deposition, and can arise from the activation and proliferation of resident fibroblasts, EMT and EndMT, macrophage-to-myofibroblast transition, recruitment and differentiation of bone marrow-derived fibrocytes, and differentiation of pericytes and mesenteric adipose tissue-derived precursors (83–85).

**Figure 2:**
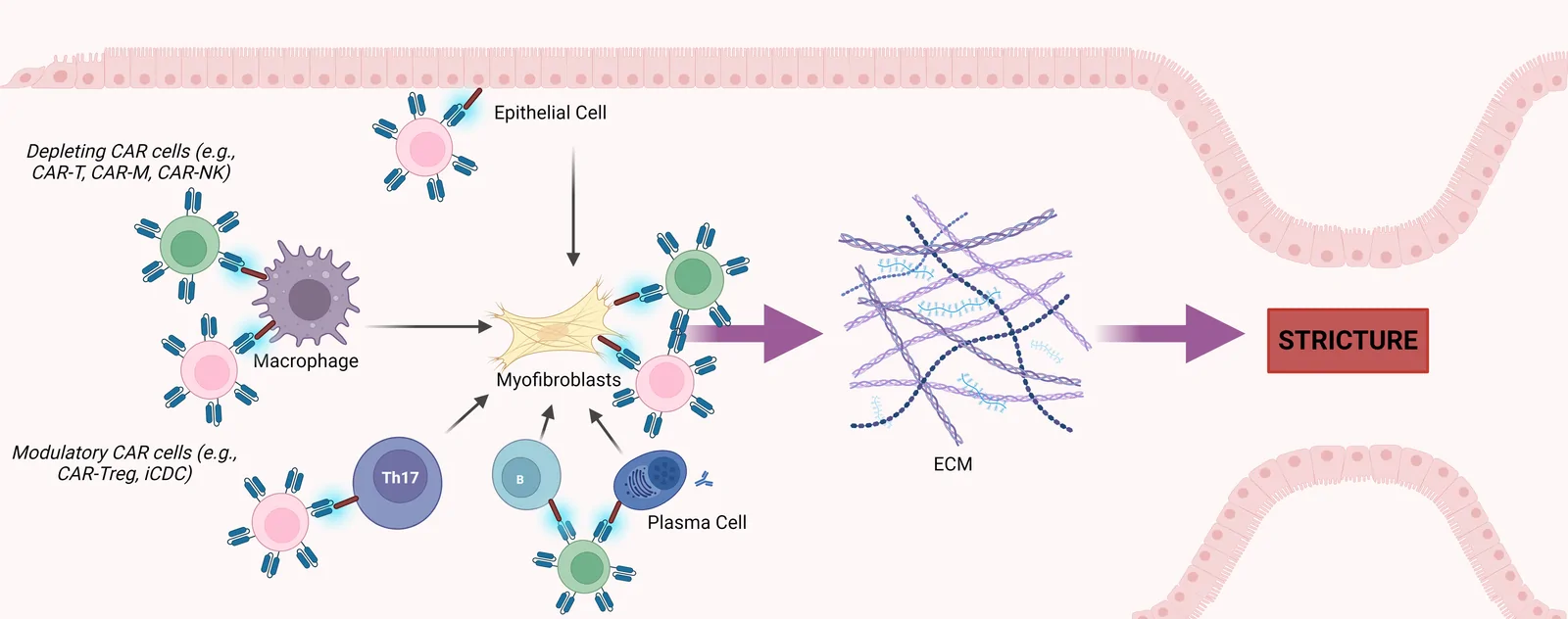
The pathogenesis of intestinal fibrosis in Crohn’s disease. Intestinal fibrosis in FSCD is driven by a multicellular network in which epithelial cells, macrophages, Th17/ILC3, and B cells collectively activate fibroblasts, the central effector cells responsible for excessive ECM deposition and stricture formation. Understanding the distinct roles of each pathogenic cell type is crucial for developing anti-fibrotic therapies, as the goal is to interrupt this multicellular cascade at multiple nodes. Created with “BioRender.com”. ECM, extracellular matrix.

Based on these mechanisms, CAR-based cell therapy for FSCD may pursue two broad therapeutic directions: immune resetting and fibrotic microenvironment editing. These directions are not mutually exclusive and may be pursued through either target-cell depletion or functional modulation, as illustrated by CAR-based approaches investigated in other fibrotic diseases. Immune resetting aims to reshape the immune compartment, potentially through the depletion of selected immune-cell populations, whereas fibrotic microenvironment editing seeks to disrupt established profibrotic networks by eliminating pathogenic cells, such as activated fibroblasts or profibrotic macrophages, and/or modulating their activity and interactions. Within the latter direction, tolerance restoration represents a related immunoregulatory objective that may be pursued using regulatory CAR cells, such as CAR-Tregs, which can suppress inflammation through mechanisms involving IL-10, TGF-β, and CTLA-4-mediated regulation. Although conceptually distinct, these approaches may converge in their effects on inflammation and tissue homeostasis.

### 3.3 Potential targets for CAR-based cell therapy in FSCD

Similar to the logic of target selection for CAR-based cell therapy in oncology or autoimmune diseases, the core consideration in target screening for FSCD lies in the specificity of candidate antigens. An ideal target should be highly expressed on specific pathogenic cells but expressed at low or undetectable levels in normal tissues. This principle aims to ensure the killing efficacy of depleting CAR cells such as CAR-T cells, reduce off-target toxicity, or enable precise regulation by modulatory CAR cells, thereby avoiding non-specific immunosuppression.

Notably, fibrosis exhibits a high degree of organ specificity, significantly influenced by local cellular heterogeneity and etiological differences. Uncritically adopting reported targets may result in insufficient efficacy or off-target risks. For example, while FAP CAR-T cells are effective in myocardial fibrosis, their protective effect in renal fibrosis is relatively weak (45). Therefore, when establishing therapeutic targets for FSCD, it is necessary to re-identify intestine-specific pathogenic cell subsets and their specific surface antigens in order to design safe and effective targeted therapies.

Unlike B cell malignancies, which possess well-established B-lineage targets such as CD19, FSCD lacks similarly well-defined targets, and target discovery relies to a greater extent on high-throughput sequencing approaches. More critically, Severe pathological remodeling in FSCD strictures frequently involves the deeper layers of the intestinal wall, particularly the submucosa and muscularis propria, and reliance solely on endoscopic biopsy specimens can easily miss the pathogenic stromal cell populations that drive fibrosis (86). Therefore, the ideal target screening strategy should not be limited to mucosal tissue alone but should incorporate surgically resected full-thickness intestinal wall specimens, utilizing single-cell omics and spatial transcriptomics to identify specific pathogenic cells and their surface antigens, thereby laying the foundation for constructing safe and efficacious CAR cell therapies.

The targets listed below, organized by the target cell population, represent candidate molecules with relatively favorable disease specificity in FSCD, selected based on the above principles (**Figure 3**). The FSCD-specific evidence level, physiological roles, safety concerns, and preferred CAR strategy for each candidate are summarized in **Table 3**.

**Figure 3:**
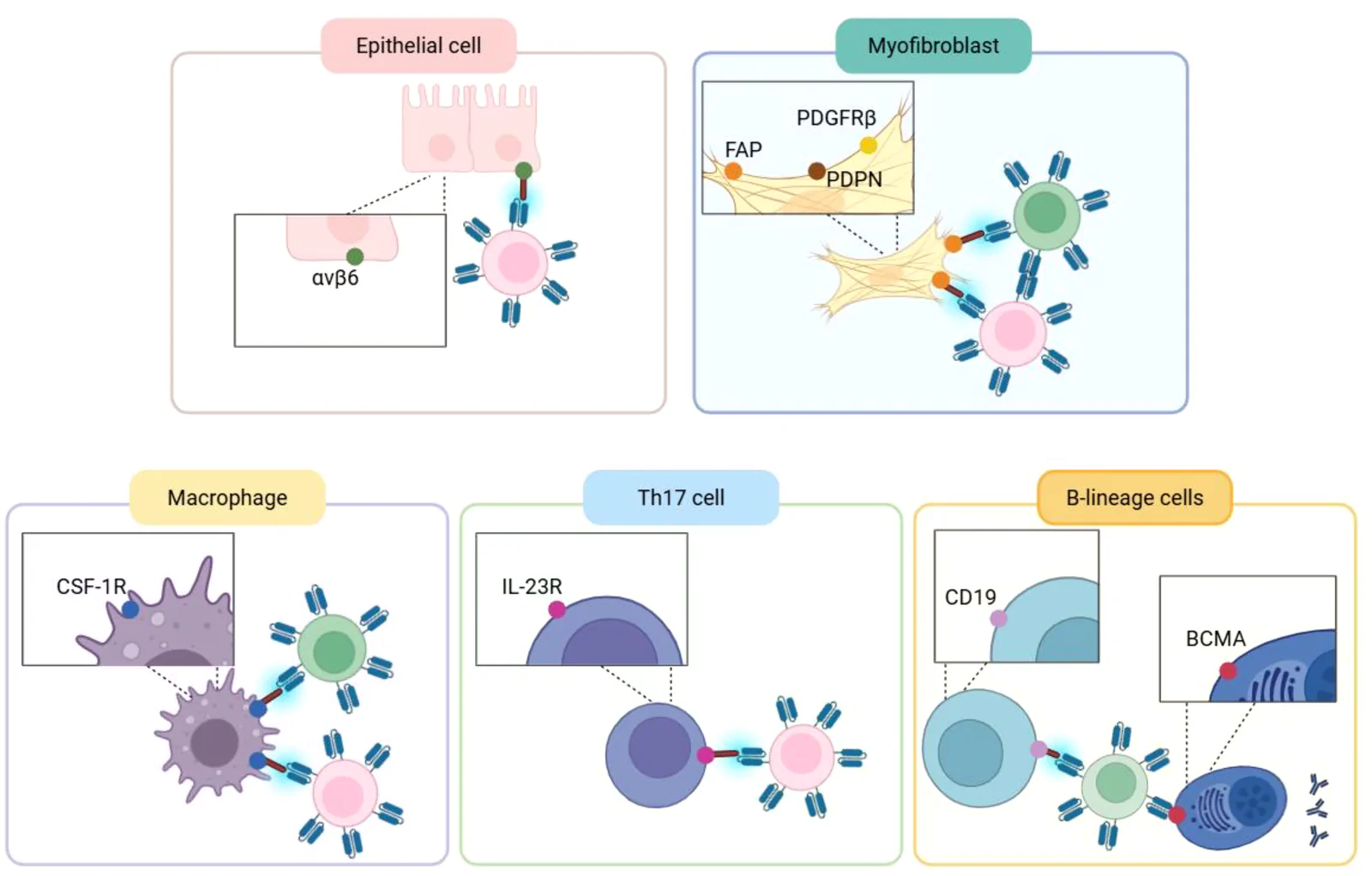
Candidate molecules for FSCD. FSCD involves multiple pathogenic cell types, each expressing distinct surface molecules that can serve as potential CAR targets. Epithelial cells express αvβ6 integrin, an upstream activator of TGF-β that drives fibrotic signaling; however, given the role of these cells in barrier maintenance, αvβ6 is better suited for modulatory rather than depleting CAR strategies. Myofibroblasts, the primary ECM-producing effector cells, express three promising targets for depleting CAR cells: platelet-derived growth factor receptor β (PDGFRβ), fibroblast activation protein (FAP), and podoplanin (PDPN). Each of these molecules is specifically upregulated on activated fibroblasts in fibrotic tissue while showing minimal expression in normal adult tissues. Macrophages, which sustain the pro-fibrotic microenvironment through secretion of TGF-β and other mediators, express colony-stimulating factor 1 receptor (CSF-1R), a key regulator of macrophage survival and polarization. Th17 cells express IL-23 receptor (IL-23R), which maintains Th17 lineage stability and pathogenic function; given the presence of protective IL-22^+^ subsets within IL-23R^+^ populations, this target is better suited for modulatory CAR cells. Plasma cells, which contribute to fibrosis through pathogenic antibodies and pro-fibrotic cytokines, express CD19 and B cell maturation antigen (BCMA), both of which have been clinically validated as depleting CAR targets in autoimmune diseases with fibrotic complications. Created with “BioRender.com”. FAP, fibroblast activated protein; PDGFRβ, platelet-derived growth factor receptor β; PDPN, podoplanin; CSF-1R, Colony-stimulating Factor-1; IL-23R, interleukin-23 receptor; BCMA, B-cell maturation antigen.

**Table 3:** Evidence map for candidate CAR targets in fibrostenosing Crohn’s disease (FSCD).

Target	Principal target cells in FSCD	FSCD target evidence	CAR therapeutic evidence	Physiological expression/function	Major on-target/off-tissue concern	Preferred CAR strategy in FSCD
αvβ6	Injured/activated intestinal epithelial cells (78, 79)	Moderate: intestinal fibrosis studies support αvβ6-TGF-β and FAK/AKT signaling (87, 88); direct FSCD stratification remains limited.	No direct CAR efficacy in FSCD; antifibrotic targetability is supported by non-CAR integrin inhibition in other organs (89–91).	Low in most quiescent adult epithelia; induced after injury; contributes to epithelial repair and intestinal barrier maintenance (92).	Direct depletion may impair mucosal repair/barrier integrity.	Modulatory rather than depleting CAR; transient/local control favored.
FAP	Activated fibroblasts/myofibroblasts (19)	Strong target-level evidence: human strictured ileum single-cell/spatial data and ex vivo inhibition link FAP+ fibroblasts to ECM production (93); human FAPI imaging supports *in vivo* detectability (94).	Antifibrotic CAR efficacy in cardiac (37), hepatic (59), and pulmonary (63) models; no direct FSCD CAR efficacy.	Activated stromal cells in injury and repair; also present in bone-marrow stromal compartments (95).	Potential impairment of repair; FAP-directed T cells caused bone toxicity/cachexia in mice (95).	Depleting strategy only with strong safety controls (transient/local/switchable); modulatory FAP-directed approaches are attractive alternatives.
PDGFRβ	Activated fibroblasts; perivascular mesenchymal cells	Moderate-to-strong target evidence: upregulated/activated in fibrotic CD tissue and linked to collagen expression (96); blockade attenuates ileal fibrosis in mice (96).	PDGFRβ CAR-T reduced renal fibrosis in mouse and kidney-organoid models (45); no FSCD CAR efficacy.	Canonical receptor on pericytes; PDGF-B/PDGFRβ signaling supports pericyte recruitment and vascular stability.	Potential vascular/pericyte injury and impaired microvascular integrity.	Requires intestine/pathogenic-subset specificity; transient or logic-gated depletion preferred over broad constitutive targeting.
PDPN	PDPN+ fibroblast-like stromal cells; lymphatic endothelium	Preliminary for FSCD: increased in IBD and stromal-like cells (97), but fibrosis-specific human validation is limited.	No antifibrotic CAR evidence in FSCD; CAR efficacy reported mainly in cancer models.	Constitutive expression in lymphatic endothelium, alveolar type I cells, glomerular podocytes and other normal tissues (98).	Broad depleting CAR may damage lymphatic, pulmonary, or renal compartments.	Not suitable for broad depletion without disease-restricted validation; consider non-depleting or combinatorial recognition.
CSF-1R	Monocytes/macrophages, including profibrotic macrophage subsets	Moderate mechanistic evidence: macrophages are implicated in intestinal fibrosis (99); CSF-1R is central to macrophage survival/homeostasis (100).	CSF-1R CAR-T has been explored in oncology (101), but no CAR antifibrotic study in FSCD or other fibrosis models.	Required for normal macrophage survival (100); CSF1R-dependent intestinal macrophages support crypt/stem-cell homeostasis (102).	Broad macrophage depletion may disturb mucosal homeostasis, repair, and host defense.	Prefer pathogenic-subset selection or temporally controlled modulation/depletion rather than pan-CSF-1R ablation.
IL-23R	Th17 cells, ILC3s and other IL-23-responsive immune cells	Preliminary for fibrostenosis: upregulated in active CD (103); fibrosis linkage is indirect and not FSCD-stratified.	IL-23R CAR-Treg shows antigen-specific suppression and tissue migration in CD models (103); no fibrosis-specific endpoint.	IL-23R signaling can support protective intestinal IL-22 responses.	Depletion may remove protective IL-22-producing populations and impair barrier repair.	Modulatory CAR-Treg favored; avoid indiscriminate cytotoxic depletion.
CD19/BCMA	B-lineage cells and plasma cells	Moderate indirect evidence: B-lineage/plasma-cell infiltration and profibrotic activity are associated with CD/FSCD (104).	Clinical CAR studies exist in CD/IBD, but no direct evidence of antifibrotic efficacy or mature-stricture reversal.	Normal B-lineage/plasma-cell antigens with established roles in humoral immunity.	B-cell/plasma-cell depletion can impair protective humoral immunity; effect on fixed fibrosis is uncertain.	Depleting strategy may support immune resetting in inflammatory/mixed disease; not a direct ECM-reversal strategy.

Target evidence and CAR therapeutic evidence are separated to avoid conflation. Evidence grading: strong, direct human FSCD full-thickness/single-cell/spatial/ex vivo data; moderate, supportive CD/IBD tissue plus mechanistic/preclinical data; preliminary, limited or indirect FSCD evidence.

#### 3.3.1 Targets on epithelial cells

αvβ6 integrin is a specific heterodimeric transmembrane receptor expressed exclusively on the surface of epithelial cells. In the vast majority of normal adult tissues, αvβ6 expression is extremely low or even undetectable. However, during tissue injury, inflammation, and fibrotic remodeling, its expression in epithelial cells can not only activate the classical TGF-β profibrotic pathway by binding to the RGD motif on LAP, but also directly activate the FAK/AKT signaling axis, driving the fibrotic process. In FSCD, evidence from multiple models of intestinal fibrosis suggests that αvβ6 contributes to disease progression (87, 88). In renal fibrosis models, the dual αvβ1/αvβ6 inhibitor Bexotegrast significantly reduced renal fibrosis and collagen deposition in mice (89). The potential of αvβ6 as an antifibrotic target has been investigated in preclinical models and clinical studies of pulmonary fibrosis. In the Phase IIa INTEGRIS-IPF study, bexotegrast showed preliminary evidence of reduced FVC decline over 12 weeks (90, 91). However, the subsequent Phase IIb/III BEACON-IPF trial was discontinued after an unfavorable risk–benefit assessment (91). These findings highlight the challenges of translating integrin-targeted antifibrotic approaches into clinical practice and underscore the importance of optimizing both target engagement and safety. Given the physiological functions of αvβ6 in epithelial injury repair and intestinal barrier maintenance (92), in FSCD, modulatory rather than depleting CAR-based cell therapy is advisable for targeting αvβ6.

#### 3.3.2 Targets on ECM-producing cells

##### 3.3.2.1 FAP

FAP is a type II transmembrane glycoprotein belonging to the serine protease family, localized to the cell membrane. It is predominantly expressed on activated fibroblasts and is largely undetectable in most normal adult tissues (37). Single-cell RNA sequencing analysis has revealed that the proportion of fibroblasts in strictured ileum is markedly higher than in inflamed ileum and nearly zero in healthy tissue, and spatial co-localization analysis further validated that FAP+ cell enrichment in the submucosa correlates positively with fibrosis severity (93). This restricted expression pattern, together with the functional role of FAP in ECM production, has made FAP one of the most extensively investigated targets for antifibrotic CAR-based cell therapy. Critically, the *in vivo* accessibility of FAP as a therapeutic target is corroborated by preclinical and prospective human imaging studies, in which FAPI-PET/CT uptake has been shown to reflect the severity of intestinal fibrosis (94, 105). FAP+ fibroblasts not only serve as a molecular marker identifying activated fibroblast subsets but are also the effector cells directly mediating excessive ECM deposition. Functional analysis has shown that these cells highly express collagen genes and fibrosis-related pathway genes, with ECM scores significantly exceeding those of other fibroblast subsets (93). That study further demonstrated *in vitro* that FAP inhibition reduces collagen deposition. Another study similarly showed that treating strictured tissue with anti-FAP antibody *in vitro* induced a decrease in type I collagen and reduced TIMP-1 production in CD strictures (106). The profibrotic effect of FAP is mediated through the regulation of the core transcription factor TWIST1, and TWIST1 knockout reverses the fibrotic phenotype (19). CAR-T therapy directly targeting FAP has shown significant efficacy in animal models. In cardiac, hepatic, and pulmonary fibrosis models, FAP-CAR-T cells reduce collagen deposition and organ dysfunction by eliminating activated fibroblasts, demonstrating the trans-organ antifibrotic potential of FAP-CAR-T cells. However, FAP is not entirely fibrosis-specific: FAP-directed T cells have recognized multipotent bone marrow stromal cells and caused cachexia and severe bone toxicity in mice, highlighting a potential off-target risk for depleting strategies (95).

##### 3.3.2.2 PDGFRβ

PDGFRβ is a receptor tyrosine kinase localized to the cell membrane. Its expression is significantly upregulated in fibrotic intestinal tissue from CD patients compared with normal intestinal tissue. Spatial transcriptomics showed significant upregulation of PDGFRB gene expression in fibrotic regions of CD patients, while enhanced PDGFRβ activity in these regions was demonstrated by protein phosphorylation assays (96). Transcriptomic data further indicate that PDGFRβ expression correlates positively with multiple collagen genes (107). Mechanistically, PDGFRβ signaling promotes fibrogenesis through multiple pathways, including GSK3β phosphorylation-dependent inactivation, leading to increased collagen synthesis (96). In parallel, PDGFB/PDGFRβ signaling induces ETS1-mediated apoptosis resistance in fibroblasts, enabling their sustained survival and continued ECM production. Furthermore, PDGFRβ signaling contributes to a positive feedback loop by inducing pro-inflammatory cytokines, while inflammatory cytokines in turn upregulate PDGFRB expression, perpetuating fibrogenesis (107). Consistent with these findings, anti-PDGFRβ neutralizing antibody attenuated ileal fibrosis in SAMP1/YitFc mice (96). Beyond the intestine, targeted delivery of IFN-γ to PDGFRβ+ myofibroblasts significantly reduced renal fibrosis (108), and anti-PDGFRβ antibody suppressed bleomycin-induced lung fibrosis (109). More directly, PDGFRβ CAR-T cells significantly reduced fibrosis and improved renal function in multiple chronic kidney disease (CKD) mouse models. In a human kidney organoid fibrosis model, human PDGFRβ CAR-T cells were similarly effective (45). In summary, PDGFRβ is specifically upregulated in fibrotic intestinal tissue and governs multiple profibrotic mechanisms in activated fibroblasts, making it a promising CAR-T target for FSCD.

##### 3.3.2.3 Podoplanin

PDPN is a mucin-type transmembrane glycoprotein, initially identified as a specific marker of lymphatic endothelium. Recent studies have demonstrated that PDPN is aberrantly expressed in both intestinal tissue and peripheral blood of patients with inflammatory bowel disease. Spatial and cellular-level analyses revealed that PDPN+ cells encompass not only lymphatic endothelial cells but also a population of fibroblast-like cells co-expressing FAP and α-SMA on their surface, possessing characteristics of pathogenic fibroblasts. Transcriptomic public database analyses further indicated that PDPN mRNA expression in intestinal tissue of inflammatory bowel disease (IBD) patients is significantly higher than in healthy controls and is particularly pronounced in the colon. Furthermore, PDPN+ cells are enriched in patients unresponsive to anti-TNF therapy, suggesting their potential as a therapeutic target in refractory IBD (97). The role of PDPN+ cells has drawn attention in fibrotic diseases. Following cardiac ischemic injury, PDPN is upregulated in various interstitial cells, including PDGFRα+ and PDGFRβ+ cells within the infarct zone, which participate in fibrosis formation and vascular remodeling during myocardial repair. PDPN is the sole ligand of C-type lectin-like receptor 2 (CLEC-2), which is expressed on immune cells such as platelets and activated monocytes/macrophages, mediating stromal-immune cell interactions. In a myocardial infarction model, PDPN-neutralizing antibody blocked this axis, reducing inflammation and improving cardiac function (110). In a pulmonary fibrosis model, PDPN is not only highly expressed in activated fibroblasts but also mediates their differentiation into myofibroblasts. The PDPN-targeting monoclonal antibody SZ168 blocked the PDPN/CLEC-2 interaction, inhibiting platelet activation and downstream RhoA/ROCK signaling, and reducing pulmonary fibrosis (111). To date, no studies have directly reported PDPN-targeting CAR-T cells in FSCD. Although PDPN-targeting CAR-T cells have validated efficacy in glioblastoma models (112), whether PDPN represents a viable CAR target in FSCD remains to be functionally tested. PDPN is constitutively expressed in several normal compartments, including lymphatic endothelium, alveolar type I cells, and glomerular podocytes (98). This distribution substantially narrows the therapeutic window for a depleting PDPN-directed CAR strategy and favors approaches that require stronger disease-restricted validation or non-depleting modulation.

#### 3.3.3 Targets on macrophages

Colony-stimulating factor 1 receptor (CSF-1R), also known as CD115 or c-FMS, is a type I single-pass transmembrane protein. CSF-1R is highly expressed on the monocyte/macrophage lineage and serves as the core regulatory molecule governing the survival, proliferation, and differentiation of these cells (100). CSF-1R is significantly upregulated on pathogenic macrophages in various fibrotic diseases and correlates closely with fibrosis severity. In chronic graft-versus-host disease (cGVHD) fibrotic skin and lung tissues, donor-derived CSF-1R+ macrophages are key drivers of fibrosis (113). Anti-CSF-1R antibody has demonstrated definitive efficacy against cGVHD fibrosis in a clinical trial (114) and received FDA approval for cGVHD treatment (115). In a renal ischemia-reperfusion-induced fibrosis model, a CSF-1R inhibitor significantly reduced renal macrophage infiltration and attenuated interstitial fibrosis. The macrophages reduced concurrently exhibited M2-like phenotypic features, whereas M1-like macrophage numbers showed no significant change (116). Further studies have shown that CSF-1R plays a critical role in macrophage polarization (117). In FSCD, CSF-1R is similarly highly expressed on intestinal macrophages, and the development and homeostatic maintenance of intestinal macrophages are highly dependent on the CSF-1/CSF-1R signaling axis (99). In the chronic inflammatory microenvironment, intestinal macrophages polarize toward an M2 profibrotic phenotype, expressing markers such as CD206 and CD163, and drive FSCD development through multiple mechanisms, including the secretion of profibrotic factors such as TGF-β1 and CTGF, induction of MMT, and inhibition of collagen degradation (99). CSF-1R inhibitors have demonstrated ameliorative effects on intestinal inflammation in IBD models. Studies have shown that CSF-1R inhibitors reduce macrophage and CD3+ T cell numbers in the colonic mucosa and lower histological inflammation scores (118). CAR-based cell therapy targeting CSF-1R has been explored in the field of oncology. Zhu et al. (101) directly utilized CSF1, the natural ligand of CSF-1R, as the recognition domain to construct CSF1-CAR. *In vitro* experiments demonstrated that these CAR-T cells not only effectively killed CSF-1R-high pancreatic cancer cells but also specifically eliminated CSF-1R+ M2 tumor-associated macrophages. However, CAR-based cell therapy targeting CSF-1R has not yet been reported in fibrotic diseases, and whether it can alleviate fibrosis progression through the elimination of pathogenic macrophages remains a gap awaiting investigation.

#### 3.3.4 Targets on Th17 cells

IL-23 receptor (IL-23R) is a type I transmembrane protein expressed on the surface of various immune cells and serves as the core receptor molecule maintaining Th17 lineage stability and pathogenic function. IL-23R is expressed at relatively low levels in the physiologically normal intestine but is significantly upregulated on immune cells in the colon and ileum of patients with active CD (103). Although that study did not separately stratify FSCD samples, given that FSCD stricture sites are characterized by chronic transmural inflammation and persistent immune activation, the sustained activation of the IL-23/IL-23R axis in this microenvironment may represent an upstream event that promotes Th17-mediated responses, which have been implicated in intestinal fibrosis (119, 120). Importantly, IL-23R is expressed by multiple cell types beyond Th17 cells, including ILCs and intestinal epithelial cells, whose roles in mucosal immunity are distinct and context-dependent (121). Given the protective contribution of epithelial IL-23R signaling to host defense and tissue restitution, as well as the incompletely defined roles of ILCs in human IBD, depletion of IL-23R^+^ cells could compromise beneficial mucosal functions, making modulatory rather than depleting CAR strategies more appropriate for this target. Researchers have already designed CAR-Tregs targeting IL-23R for the treatment of CD. Functional experiments demonstrated that IL-23R-CAR Tregs are specifically activated upon encountering IL-23R and effectively suppress effector T cell proliferation and dendritic cell maturation, offering a potential cellular immunotherapy strategy to induce immune tolerance in FSCD (103).

#### 3.3.5 Targets on B cells

CD19 is a type I transmembrane glycoprotein belonging to the immunoglobulin superfamily, specifically expressed on the surface of the B cell lineage. CD19 is expressed from the early pre-B cell stage onward, persisting through memory B cell and plasmablast stages, with expression downregulated in terminally differentiated plasma cells (122). In the FSCD process, the B cell lineage can serve both as a source of pathogenic IgG4 antibodies and as a producer of fibrosis-driving cytokines. One study identified that the infiltration density of IgG4+ plasma cells in the intestinal wall is significantly positively correlated with histological fibrosis scores, suggesting that these terminally differentiated plasma cells are important effector cells driving transmural fibrosis (104). Chen et al. (82) further found that in the intestinal inflammatory environment of CD patients, the direct differentiation pathway of activated memory B cells (MBCs) to plasma cells (PCs) is aberrantly reinforced, meaning that MBCs more readily bypass secondary germinal center selection. That study also identified a population of gut-resident-like MBCs and found that they exacerbate intestinal fibrosis through the high expression of the profibrotic factor amphiregulin. Therefore, targeting the CD19 molecule broadly expressed across all B cell subsets, and conducting a deep depletion via CAR-T cells, can theoretically simultaneously block upstream profibrotic memory B cell activation and expansion, and deplete plasmablasts and some plasma cells producing IgG4 antibodies, thereby curbing fibrostenotic progression. Given that terminally differentiated plasma cells generally downregulate CD19 expression, CD19-targeted strategies alone may leave behind a population of long-lived plasma cells as a potential source of relapse (123). A dual-targeting approach against CD19 and BCMA, which enables more complete coverage of B lineage cells and plasma cells, therefore represents a more optimal strategy. Currently, a BCMA-CD19 dual-targeting CAR-T cell therapy (NCT07544160) is under clinical evaluation for refractory IBD, and a universal CD19/BCMA-targeting CAR-T cell therapy (NCT07309744) is being evaluated for moderate-to-severe CD and UC, which will directly test the feasibility of these therapeutic strategies *in vivo*.

### 3.4 Selecting a CAR platform for FSCD

The selection of a CAR platform for FSCD requires alignment between the dominant pathological process, the cellular compartment to be targeted, and the intended therapeutic outcome. Unlike inflammation-dominant CD, FSCD involves variable contributions from immune activation, activated stromal cells, ECM accumulation, and structural remodeling of the bowel wall. Platform selection must therefore consider not only whether engineered cells can reach and engage the relevant targets within fibrotic lesions, but also whether their effector functions are appropriate for the disease stage without compromising intestinal homeostasis. **Table 4** therefore compares the principal characteristics and potential therapeutic roles of these platforms while distinguishing their general biological properties from their evidence base in FSCD.

**Table 4:** Comparative characteristics and intended therapeutic roles of CAR-T, CAR-M, CAR-NK, and CAR-Treg platforms in FSCD.

Platform	Main mechanism	Tissue trafficking and lesion penetration	Expected persistence of CAR activity	Manufacturing considerations	Major safety concern	Potential therapeutic role and disease stage	Direct FSCD evidence
CAR-T	Antigen-specific cytotoxic depletion of target cells (37); can be engineered for transient or persistent activity (58).	Requires extravasation, chemokine-guided trafficking and ECM penetration (124, 125); dense/stiff matrix may limit access to deep lesions (125).	Typically high with conventional ex vivo products (126); lower with transient *in vivo* mRNA approaches (58).	Mature ex vivo manufacturing ecosystem but costly/slow (127); *in vivo* generation may simplify production (58).	CRS/ICANS (128); on-target/off-tissue injury; prolonged depletion of physiologic target cells (95).	Prevent progression or suppress ongoing fibrogenesis by depleting pathogenic fibroblasts/immune cells; reversal of mature strictures unproven.	None for FSCD.
CAR-M	Target recognition with phagocytic/cytotoxic activity plus innate tissue remodeling and cytokine functions (129).	Potentially favorable tissue infiltration and matrix remodeling (41, 43), but dense ECM and macrophage plasticity remain barriers.	Intermediate/variable; generally less durable than conventional CAR-T (41, 43).	Myeloid engineering and expansion are more complex; *in vivo* engineering is emerging.	Pro-inflammatory/profibrotic repolarization; off-tissue phagocytic injury; cytokine toxicity.	Suppress ongoing fibrogenesis and remodel the fibrotic niche; may be attractive for transmural lesions, but mature-stricture reversal is unproven.	None for FSCD.
CAR-NK	Antigen-specific cytotoxicity with innate killing (127); potential allogeneic/off-the-shelf use.	Faces the same vascular/ECM penetration barriers as other lymphocytes; shorter persistence may limit sustained activity.	Usually shorter than CAR-T (127).	Allogeneic manufacture is attractive, but expansion, cryopreservation, and persistence remain challenges (127).	On-target/off-tissue cytotoxicity; limited persistence may reduce but not eliminate systemic toxicity.	Shorter, controllable depletion to prevent progression or suppress active fibrogenesis; not a direct ECM-removal platform.	None for FSCD.
CAR-Treg	Antigen-specific immune suppression, tolerance restoration, and local regulation rather than direct cytotoxic ECM removal (103).	Can home to antigen/inflammatory signals (103); tissue access remains important, but therapeutic effect does not require direct matrix phagocytosis.	Variable; durable benefit depends on lineage stability and local survival (130, 131).	Requires high-purity Treg isolation/expansion and maintenance of FOXP3/lineage stability (130, 132).	Phenotypic instability (131), systemic immunosuppression, or unintended suppression of protective immunity.	Best aligned with inflammatory/mixed disease to prevent progression or suppress inflammation-driven fibrogenesis; not expected to reverse mature ECM directly.	IL-23R CAR-Treg studied in CD without fibrosis-specific endpoints; none for FSCD reversal.

The therapeutic intent column distinguishes prevention of progression, suppression of ongoing fibrogenesis, and reversal of mature strictures. No platform currently has direct evidence demonstrating reversal of mature FSCD strictures.

### 3.5 Whether CAR therapy can reverse established fibrostenosis

A distinction should be made between suppressing ongoing fibrogenesis and reversing established fibrostenosis. Eliminating pathogenic fibroblasts could reduce ongoing collagen synthesis, but this alone is unlikely to be sufficient to reverse a mature stricture. Established FSCD contains the contraction of the submucosa, excessive accumulation of collagen and structural remodeling of the bowel wall, including muscularis propria hypertrophy and hyperplasia. Therefore, reversal of such lesions would require not only the cessation of further matrix deposition but also effective ECM clearance or remodeling and recovery of bowel-wall architecture and mechanical function.

Current evidence supports the targetability of fibrogenic pathways in intestinal strictures, but it does not yet establish that CAR-based therapy can reverse mature fibrostenosis. For example, human FSCD tissues have shown that FAP inhibition can reduce collagen production ex vivo (106), which supports targetability of ongoing fibrogenesis, but does not demonstrate that CAR-based therapy can reverse mature fibrostenosis *in vivo*.

The safety trade-off is equally important because stromal and immune target populations also contribute to tissue homeostasis and repair. Fibroblasts participate in wound healing, and FAP-directed T cells have caused bone-marrow stromal toxicity and cachexia in preclinical models (95). Likewise, αvβ6 contributes to epithelial barrier maintenance. These observations argue against assuming that deeper depletion will necessarily produce better structural outcomes; spatially restricted, transient, or modulatory strategies may be preferable when target-positive populations contain protective subsets.

Accordingly, the most defensible near-term therapeutic objectives for CAR-based approaches in FSCD are to prevent fibrosis progression and suppress ongoing fibrogenesis. True reversal of mature strictures remains unproven and should be treated as a distinct, higher bar that requires demonstration of durable structural improvement in transmural imaging and, where feasible, histopathology.

## 4 Exploration and current status of CAR-based cell therapy for FSCD

### 4.1 CAR-based cell therapy applications in CD

Compared with other diseases, the application of CAR-based cell therapy in FSCD remains in its infancy. To date, no CAR-based cell therapy studies specifically targeting FSCD exist; however, exploratory efforts have been undertaken in the field of CD. Based on differences in mechanism of action and target, current CAR-based cell therapy studies in CD can be broadly categorized into three major pathways: T cell depletion, B cell/plasma cell depletion, and the use of CAR-Tregs.

A Phase I clinical trial (NCT05239702) is currently evaluating the safety, tolerability, and preliminary efficacy of CD7-CAR-T cells in patients with autoimmune diseases including CD and ulcerative colitis. CD7 is a transmembrane glycoprotein belonging to the immunoglobulin superfamily, expressed on the surface of most mature peripheral blood T lymphocytes, thymocytes, natural killer cells, and immature hematopoietic progenitor cells (133). CD7-targeting CAR-T cells hold the potential to suppress intestinal immune responses by eliminating pathogenic T lymphocytes. However, this study enrolls a broad disease spectrum, with efficacy evaluated primarily using endpoints such as overall response rate.

Two clinical studies on CAR-T therapy targeting the depletion of B cells and plasma cells have entered the early validation stage. A not-yet-recruiting Phase I/IIa study (NCT07544160) plans to evaluate the safety and efficacy of a BCMA/CD19 dual-targeting compound CAR-T cell armored with an IL-15/IL-15sushi complex in patients with relapsed/refractory IBD. The endpoint design of this study specifically incorporates deep evaluative indicators, including the Crohn’s Disease Activity Index (CDAI), endoscopic remission, histological remission, drug-free remission, and fecal calprotectin levels, capable of directly reflecting the ability of CAR-T therapy to control inflammation and improve intestinal mucosal lesions. Another not-yet-recruiting study (NCT07309744) plans to employ universal CD19/BCMA CAR-T cells and has established a dedicated treatment cohort for CD, with three pre-specified dose groups to explore the optimal biological dose. Key observational indicators include CDAI, histological remission, CRP levels, and fecal calprotectin levels. The aforementioned two studies will directly evaluate whether B cell/plasma cell-depleting CAR-T cells can infiltrate the intestinal mucosa, eliminate local pathogenic immune cells, and restore intestinal immune homeostasis. Their endpoint designs are also optimized for refined assessments of intestinal inflammation control and mucosal healing.

In addition, studies have explored CAR-Treg therapy targeting IL-23R for the treatment of CD. In CD, IL-23, through IL-23R signaling, drives Th17 cell differentiation and expansion, promotes the production of pro-inflammatory factors such as IL-17, IL-22, and IFN-γ, and directly mediates intestinal inflammatory responses (134). On the other hand, IL-23 can directly act on IL-23R on the surface of Treg cells, suppressing their stability and function, thereby disrupting the balance between pro-inflammatory and anti-inflammatory immunity and exacerbating the inflammatory process. Furthermore, IL-23 can amplify the inflammatory response by acting on various innate immune cells (135). Based on the above mechanisms, CAR-Tregs targeting IL-23R can theoretically not only specifically recognize and suppress the activation and proliferation of IL-23R+ T lymphocytes and antigen-presenting cells but also replenish Treg numbers to restore immune homeostasis. In a recent study, researchers constructed CAR-Tregs targeting IL-23R and demonstrated that they specifically inhibit T lymphocytes and antigen-presenting cells *in vitro* and specifically migrate to IL-23R-expressing tissues in a mouse model, providing a highly promising new strategy for the treatment of CD (103).

Importantly, these studies have not directly evaluated whether CAR-based interventions can reverse or otherwise therapeutically modify established fibrosis. Future clinical studies of CAR-based therapies specifically targeting FSCD should therefore build on established clinical and endoscopic assessments in Crohn’s disease while incorporating FSCD-specific measures and endpoints to evaluate their effects on intestinal fibrosis and stricture-related outcomes.

### 4.2 Clinical trial endpoints for CAR-based cell therapy in FSCD

#### 4.2.1 Inflammation control

Inflammation control is the traditional primary goal of CD treatment and may also serve as a prerequisite for subsequent fibrosis improvement. In clinical trials for luminal CD, this is a well-established treatment objective (136). However, in FSCD, the correlation between these indicators and the degree of fibrostenosis is relatively weak. FSCD patients may still present with obstructive symptoms due to fixed fibrotic strictures even during clinical remission without signs of active inflammation (137). Therefore, inflammation control may serve as an adjunctive endpoint or early pharmacodynamic evidence but is not in itself equivalent to antifibrotic efficacy.

#### 4.2.2 Mucosal healing

According to the CORE-IBD consensus, mucosal healing has been established as one of the core outcome measures in randomized controlled trials for luminal CD. In FSCD trials, for mixed strictures with concurrent active inflammation, mucosal healing remains an important efficacy dimension. However, for fixed strictures dominated by transmural fibrosis with a relatively intact mucosal surface, the clinical significance of mucosal healing as a primary endpoint may be limited, given that Simple Endoscopic Score for Crohn’s Disease (SES-CD) itself focuses on ulcer size and extent and lacks the capacity to assess transmural lesions such as bowel wall thickening and luminal narrowing. In such cases, cross-sectional imaging should be incorporated to assess transmural changes (136).

#### 4.2.3 Transmural healing

Transmural healing has not yet been incorporated into the core outcome set for IBD randomized controlled trials (136). However, under current technical conditions, it should be considered a candidate primary efficacy endpoint for FSCD trials. MRE and IUS can serve as objective cross-sectional imaging tools for assessing transmural healing (138, 139).

#### 4.2.4 Fibrosis improvement

Fibrosis improvement should be positioned as a core exploratory endpoint in FSCD trials. Although a 2022 international consensus reached agreement on standardized items for histological assessment, these are still under validation (140). Furthermore, specimen acquisition depends on surgical resection, limiting its applicability to opportunistic cohorts only. For non-invasive assessment, CTE- and MRE-based parameters validated by the STAR consortium, including stricture length, maximal bowel wall thickness at the stricture site, and maximal pre-stenotic dilatation, can serve as first-line imaging metrics for quantifying fibrosis improvement, given their established correlation with stricture severity (76, 77). Additionally, FAPI-PET/CT can visualize fibrotic activity by targeting activated fibroblasts and is currently in the clinical validation stage (75).

### 4.3 Translational considerations for CAR-based cell therapy in FSCD

#### 4.3.1 Technical constraints

Conventional CAR-based cell therapy requires genetic modification using viral vectors, which carries the risk of genomic integration (141), as well as high production costs and long manufacturing timelines (142). Additionally, this therapy routinely requires lymphodepleting preconditioning to help cell engraftment, a process that itself may increase the risks of infection and immune dysregulation, and necessitates inpatient monitoring of patients (143). Compared with oncology patients, FSCD patients have a lower risk tolerance, demanding that CAR therapies for FSCD achieve a markedly higher safety threshold than oncology indications.

#### 4.3.2 Clinical safety constraints

From a clinical safety perspective, CAR-based cell therapy carries risks of both immune activation-related toxicities, principally cytokine release syndrome (CRS) and immune effector cell-associated neurotoxicity syndrome (ICANS), and target recognition-related adverse events arising from off-target effects. In the case of FAP, FAP plays an important role in normal wound healing, and conventional CAR cells with prolonged *in vivo* persistence may interfere with the normal wound healing process (144). These concerns also have implications for CAR-cell design in FSCD. Given that FSCD is a chronic, non-malignant condition, the acceptable risk threshold for CAR-based therapy is likely to be lower than that for life-threatening malignancies, necessitating a particularly stringent safety profile and careful risk–benefit assessment (145). Future approaches may need to incorporate mechanisms that allow therapeutic activity to be restricted to appropriate cellular contexts or terminated when necessary. Bioengineering strategies explored for non-malignant diseases include conditional activation systems, such as synNotch-based designs, and suicide genes that enable the elimination of engineered cells if serious toxicity occurs (146). Such approaches may offer ways to improve the controllability of CAR-cell activity.

Furthermore, the application of CAR-T cell therapy in the intestine, an immunologically active organ, may provoke severe immune-related adverse events. An FDA Adverse Event Reporting System-based pharmacovigilance study (147) demonstrated that BCMA-targeted CAR-T therapy is significantly associated with the risk of immune-mediated colitis (IMC). In contrast, the reporting risk of IMC associated with CD19-targeted CAR-T therapy, although not reaching statistical significance, showed a lower trend. Among CAR-T-related colitis cases, 32% required hospitalization, 17% had a fatal outcome, and 12% of cases were concomitant with CRS. These data suggest that the impact on intestinal immunity differs substantially among CAR-T therapies targeting different antigens. Therefore, when extending CAR therapy to non-oncological indications such as FSCD, careful evaluation of target selection, intestinal immune toxicity risk, and the overall tolerability of patients is warranted.

#### 4.3.3 Alternative CAR-cell generation and manufacturing strategies

The technical constraints of ex vivo CAR-T production and potential risks of lymphodepletion have spurred interest in direct *in vivo* generation approaches. Based on the delivery platform, it is currently primarily categorized into lipid nanoparticle (LNP)-based transient expression platforms and lentiviral vector-based platforms. Lentiviral vector-based platforms with integrating payloads offer advantages in oncological indications requiring durable, high-efficiency CAR-T cell activity, whereas LNP platforms, characterized by transient expression, non-integrating properties, and the feasibility of repeat dosing, hold greater potential for indications with higher safety requirements, such as autoimmune diseases. However, *in vivo* CAR technology does not eliminate clinical safety risks such as off-target toxicity, CRS, and ICANS, and it also introduces new challenges. For example, LNP-RNA-based platforms may provoke acute infusion reactions and anti-drug antibodies due to the immunogenicity of both the LNP components and the RNA payload, thereby compromising the safety of repeat dosing (148). Beyond *in vivo* CAR generation, allogeneic “off-the-shelf” CAR-cell products represent another approach to reducing the manufacturing burden associated with individualized autologous therapies (146). Nevertheless, the suitability of these approaches for FSCD will depend on the ability to achieve appropriate CAR-cell specificity, control the duration of therapeutic activity, and minimize treatment-related toxicity. For allogeneic off-the-shelf products, host immune rejection and compatibility will also need to be considered.

#### 4.3.4 Engineering strategies to improve tissue access and local CAR-cell function

A major challenge for CAR-based therapy in FSCD is whether the engineered cells can effectively access pathogenic populations located within the deep layers of the intestinal wall because FSCD particularly involves the remodeling of submucosa and muscularis propria (149). Several engineering approaches have been explored in solid-tumor research, including cell-intrinsic modifications to improve trafficking or function, microenvironmental remodeling, and tissue-directed delivery, and may therefore provide useful insights for future FSCD research (150).

For intravenously administered CAR-T or CAR-NK cells, tissue access requires sequential processes including vascular trafficking, endothelial adhesion, extravasation, chemokine-guided migration, and movement through the interstitial matrix. Studies in solid tumors have demonstrated that impaired chemokine–receptor matching, abnormal vascular structures, and dense ECM accumulation can substantially limit CAR-cell infiltration and local persistence (150). Although FSCD is biologically distinct from solid tumors, these findings provide a mechanistic framework for considering similar accessibility barriers in intestinal fibrotic lesions.

Cell-intrinsic modifications that could be adapted from solid-tumor research to FSCD include chemokine receptor engineering and matrix degradation. In solid-tumor models, CAR-T cells have been engineered to express chemokine receptors that match chemokines enriched in the tumor microenvironment (TME), thereby enhancing chemokine-directed migration and tumor infiltration (124, 151). Applying this strategy to FSCD requires consideration of the spatial distribution of candidate ligands relative to the pathological cell populations that CAR cells are intended to reach, rather than simply chemokine abundance in bulk strictured tissue. Spatial transcriptomic analyses of FSCD have identified inflammatory fibroblasts expressing chemokine-related genes, including CXCL1, CXCL5, CXCL6, and CXCL13. However, these fibroblasts are spatially associated with damaged epithelium, and the chemokines they express may not necessarily guide engineered CAR cells into deeper bowel-wall layers, such as the submucosa or muscularis propria (152). Further investigation is therefore needed to identify chemokine–receptor axes associated with pathological cell populations localized to these deeper layers, such as collagen-high fibroblasts (152). CAR cells can also be engineered to enhance tissue penetration through matrix degradation. For example, engineering CAR-T cells to express heparinase (HPSE) has been shown to enhance their infiltration and antitumor activity in solid-tumor models (153). In FSCD, where fibrillar collagens, particularly types I and III, are major structural components ECM, engineering CAR-T cells to express ECM-degrading enzymes, such as MMPs, represents a potential cell-intrinsic strategy. Supporting this concept, a study demonstrated that CAR-T cells engineered to secrete MMP-8 exhibited improved tumor infiltration and antitumor activity in xenograft mouse models (154).

The gut microbiota, which also represent a disease-relevant factor in the pathogenesis of FSCD, may become a potential source of strategies for modulating the fibrotic microenvironment. Adherent-invasive Escherichia coli (AIEC)-derived metabolites have been implicated in intestinal disease progression, including profibrotic interactions between macrophages and fibroblasts (155). These findings suggest that microbial products may contribute to the pathological microenvironment of fibrostenotic lesions. Beyond their potential role in fibrogenesis, microbial signals may also influence the recruitment of therapeutic cells by altering local immune-cell states and chemokine production. A preclinical solid-tumor study showed that intratumoral administration of Escherichia coli MG1655 promoted vascular normalization and macrophage-derived CCL5 production, thereby facilitating the infiltration of CAR-T cells (156). In parallel, engineered bacterial biomaterials, including live bacteria and acellular platforms such as outer membrane vesicles, have been investigated as approaches to remodel the local immune microenvironment and augment adoptive immune-cell therapy (157). Although these studies were conducted in tumors rather than intestinal fibrosis, they illustrate how bacterial interventions may influence not only the local tissue environment but also the access and activity of therapeutic immune cells.

In solid tumors, local injection is the major strategy for improving cell delivery (150). In FSCD, this approach could potentially be adapted to endoscopic delivery. A study in a rat model of colitis provides a preclinical precedent for endoscopic submucosal injection of mesenchymal stromal cells (MSCs) (158). More directly, endoscopic intramural injection of MSCs into the submucosa of CD strictures has been explored in a phase I–II clinical study (159).

Future studies should determine whether these strategies can improve CAR-cell access to pathogenic populations in the deeper bowel wall and establish whether enhanced tissue access translates into meaningful changes in intestinal fibrosis.

## 5 Conclusion

CAR-based cell therapy offers a potential means of intervening fibrosis. Evidence from cardiac, hepatic, pulmonary, and renal fibrosis demonstrates that engineered cells can target fibrosis-associated populations or alter the local tissue environment. However, at present, the rationale for CAR-based therapy in FSCD rests on the convergence of intestinal pathobiological evidence and proof-of-concept findings from other fibrotic settings, rather than on demonstrated FSCD-specific antifibrotic efficacy.

The choice of a CAR-based strategy should be guided first by disease stage and the pathological processes that the intervention is intended to address. In inflammation-dominant disease, controlling inflammation may help limit subsequent fibrotic progression, whereas in established fibrostenotic disease, ongoing fibrogenesis and structural remodeling may require more direct intervention. Strategy selection should therefore be based on the therapeutic objective at a given disease stage and the biological properties of the target-cell population, including its contribution to pathological remodeling and its physiological or reparative functions. Target-cell depletion and functional modulation should be considered complementary rather than mutually exclusive approaches, with their suitability determined by the intended therapeutic effect and the risks associated with disrupting the target population. And the choice of molecular target must also be considered alongside lesion accessibility, CAR-cell platform, and safety.

Clinical translation will require coordinated advances in outcome assessment, safety control, and tissue accessibility. Future clinical studies should use established Crohn’s disease assessments alongside measures suited to FSCD. At the same time, the risks of immune-mediated toxicity and on-target effects on cells involved in normal tissue maintenance must be weighed against the anticipated therapeutic benefit, particularly in this non-malignant condition. Strategies to improve CAR-cell delivery and function within the deeper layers of the intestinal wall also require validation in fibrotic lesions.
